# Effect of Ginger Powder on Production Performance, Antioxidant Status, Hematological Parameters, Digestibility, and Plasma Cholesterol Content in Broiler Chickens

**DOI:** 10.3390/ani12070901

**Published:** 2022-03-31

**Authors:** Hanan Al-Khalaifah, Afaf Al-Nasser, Tahani Al-Surrayai, Hanan Sultan, Dalal Al-Attal, Rawan Al-Kandari, Haya Al-Saleem, Aisha Al-Holi, Fatma Dashti

**Affiliations:** Environment and Life Sciences Research Center, Kuwait Institute for Scientific Research, P.O. Box 24885, Safat 13109, Kuwait; anasser@kisr.edu.kw (A.A.-N.); tsuraia@kisr.edu.kw (T.A.-S.); hsultan@kisr.edu.kw (H.S.); dattal@kisr.edu.kw (D.A.-A.); rkandari@kisr.edu.kw (R.A.-K.); hsaleem@kisr.edu.kw (H.A.-S.); aholi@kisr.edu.kw (A.A.-H.); fadashti@kisr.edu.kw (F.D.)

**Keywords:** antioxidant, broilers, ginger, hematological parameters

## Abstract

**Simple Summary:**

Chicken meat is a popular food item all over the wrld. Improving the nutrition of broilers is important for producing high-quality broiler meat. The inclusion of natural effective ingredients, such as ginger, in the diet of broilers did not adversely affect the palatability of the diet, nor did it cause anemia in the broilers. Rather, ginger enhanced the oxidative status and growth rate of broiler chickens.

**Abstract:**

The effect of dietary ginger powder on the production performance, digestibility, hematological parameters, antioxidant status, dietary oxidation stability, and plasma cholesterol content of broiler chickens was investigated. Ginger powder was included in the diet at 0, 5, 10, or 15 g/kg. Total antioxidant capacity and malondialdehyde in sera samples, superoxide dismutase activity, glutathione peroxidase, catalase, and malondialdehyde in liver samples, and the peroxide value and acid value of the stored diets were evaluated. The results showed that ginger inclusion significantly improved antioxidation indices in broiler sera and liver. Total body weight gain in ginger-supplemented birds was higher than that of control birds (*p* < 0.048). Supplementing the broiler chickens with ginger powder reduced total feed consumption (*p* < 0.031). White blood cell counts and the percentage of heterophils in the blood were increased in birds that received ginger supplementation (*p* < 0.001). The inclusion of ginger in the diet improved dry matter digestibility, crude protein utilization, crude fiber utilization, and ether extract utilization. In addition, blood cholesterol, triglyceride, and very low-density lipoprotein levels were decreased (*p* < 0.001), and high-density lipoprotein and levels were increased, following the inclusion of ginger in the diet (*p* < 0.001).

## 1. Introduction

There is interest in elevating the production performance of broiler chickens using effective nutritional additives in the feed rations, especially after the COVID-19 crisis. Medicinal plants are used as natural feed additives in poultry diets to enhance the performance, anti-oxidative status, and immune response of chickens [1,2,3,4,5,6,7,8]. One of these additives is ginger powder. Ginger is the rhizome of the plant *Zingiber officinale*. It belongs to the family *Zingibeaceae*, which includes aromatic herbs with fleshy, tuberous or non-tuberous rhizomes, that often have tuber-bearing roots [9]. It has long served as a popular culinary and traditional medicinal herb. Ginger contains several effective compounds, such as gingerol and gingerdione that exert strong antioxidant activity. In addition, it has antibacterial properties and is immunomodulatory in laboratory animals [10,11,12,13]. Plant-derived additives used in animal feed to improve production performance are known as phytogenic feed additives, and ginger is one such additive [14]. Ginger powder has lipid-reducing effects and can also be used as a growth promoter. When included in chicken feed, it has properties similar to those of antibiotics. These natural feed additives lower enteric pathogen microbial loads and improve nutrient digestion and absorption, which improve poultry production and broiler performance [15].

Antioxidants can impact the health status of poultry [16]. The inclusion of ginger in the diet at 5–6 g/kg is thought to increase total protein and lower cholesterol concentration. Studies have also found that ginger in chicken feed enhances immunity against Newcastle disease and bacterial bursal infections [17]. Because of its antioxidant properties, and ability to enhance immune function and inflammatory responses, ginger can improve both chicken production performance and the immune system [18]. Furthermore, Sahoo, Mishra [19] reported that feed rations supplemented with ginger, either alone or with 1% turmeric, significantly enhanced the antioxidative status and gut health of broiler chickens. An, Liu [18] investigated the effect of ginger supplementation on antioxidative indices in broiler chickens. The results of their study revealed that neither the activity of glutathione peroxidase nor total antioxidant capacity were affected by ginger extract supplementation, but plasma dismutase activity and malondialdehyde (MDA) content were significantly decreased. In addition, the antioxidant stability of the feed ration was increased. 

The effects of ginger have also been investigated in laying hens. For example, Wen, Gu [20] reported increased superoxide dimutase (TSOD) activity, and decreased yolk MDA and cholesterol content in hens supplemented with ginger, compared to control hens. These findings were consistent with those of Zhao, Yang [21], who found that layers fed a diet rich in ginger powder had a reduced concentration of MDA and increased TSOD activity in the yolk. In addition, Yang, Ding [22] observed increased blood antioxidant enzymes and improved egg quality in ginger root-fed laying hens.

In addition to its effect as an antioxidant, several research studies have reported that poultry diets supplemented with ginger powder have positive effects on broiler performance. As a natural feed additive, ginger may have great benefit and value in poultry nutrition—especially for broilers—due to its antibacterial, anti-inflammatory, antioxidant, antiseptic, antiparasitic, and immunomodulatory properties [23]. Ginger is a natural plant that can be used as a phytobiotic to improve the performance of broilers. This improved performance may be attributed to two types of digestive enzymes found in ginger—protease and lipase—which are part of the plant’s natural protective mechanisms [24]. Diets enriched with ginger may have the potential to improve production performance and modulate the biological properties of the blood in broiler chickens. 

Sa’aci, Alabi [25] studied the effect of aqueous ginger extract (AGE) on growth, nutrient digestibility, and the economy of Marshal broiler chicks. The AGE supplementation of the diet had no effect on dry matter, crude protein, ether extract, or nitrogen-free extract, but crude fiber digestibility was significantly affected. 

Shewita and Taha [17] reported that ethanolic extract of ginger significantly lowered serum total cholesterol and triglyceride levels and increased high-density lipoprotein (HDL) cholesterol levels, preventing tissue damage due to lipid peroxidation. It also showed lipid-lowering activity. In their study on broiler chicks, the authors fed the chicks diets supplemented with 2, 4, and 6 g/kg ginger powder and observed that serum very low-density lipoprotein (VLDL) and triglyceride levels were reduced significantly in all ginger-supplemented groups. Another study used ginger (5, 10, and 15 g/kg ginger powder) and thymol (200 and 400 mg/kg) as feed supplements in the diet of broiler chicks, which led to significantly decreased levels of serum total cholesterol and triacylglycerol [26]. 

The aim of this study was to investigate the effects of ginger on the antioxidant status, production performance, and hematological parameters of broiler chickens fed a ginger-enriched diet, as well as the oxidation stability of ginger-supplemented feed. The hypothesis was that ginger powder would improve the aforementioned parameters but would have dose-dependent effects. Although previous studies have investigated the effect of ginger on productive performance parameters in broiler chickens, relatively limited data exist in the literature on the direct effects of ginger on the antioxidative status of blood and liver tissues in these birds and the effect on the oxidative stability of the stored diets at different ginger levels.

## 2. Materials and Methods

### 2.1. Chickens, Experimental Design, and Diet

This research study was approved by the department committee of the Environment and Life Sciences Research Center in Kuwait Institute for Scientific Research under project No. FA157K (2017). These procedures and protocols followed the official animal welfare guidelines and regulations encoded with reference No. PMO/PV/RP/032/2017. This protocol recommends humane treatment of experimental animals with no pain, stress, or harm. The vitality rate was 100% and no abnormal signs were observed during the experiment. 

Fresh ginger (*Zingiber officinale*) roots were purchased from a reliable local supermarket, originated from India. The roots were washed, sliced, freeze-dried, and milled into a powder that was used in the broiler feed rations. This study used 1-day-old, male, Cobb 500 broiler chicks that were vaccinated against infectious bronchitis and Newcastle disease. Water and feed were provided ad libitum. Four experimental diets/treatments were used, with 0, 5, 10, or 15 g/kg ginger in the diet. For each treatment, 340 birds were randomly housed in a battery cage with five levels. Each level contained 17 birds, for a total of 85 birds in the battery. This density provided a space of 0.05 m^2^/bird. Each level was considered as a replicate (total of 5 replicates). The broiler chicks were fed a corn/soy-based diet that met Cobb 500 guidelines [27]. The chicks received a starter diet from hatching until 7 days of age, a grower diet from 8 to 21 days of age, and a finisher diet from 22 to 35 days of age. All diets were prepared as needed. Diet formulation, as well as chemical analyses of the control diet and ginger supplement are shown in Table 1. The control birds received no ginger. The ambient temperature for the broilers was kept at 30 °C for 14 days, and then gradually reduced to 21 °C by 21 days. A proximate analysis of the ginger was performed for crude protein, ash, dry matter, and crude fiber.

### 2.2. Sample Collection

At 5 weeks of age, blood samples were collected from the branchial vein of birds in vacutainer tubes (K2EDTA). Blood samples were collected from five chickens from each treatment, and 8–10 mL of blood was collected in each tube. Meat tissue and liver samples were also collected from five chickens per treatment. Analysis was completed in triplicates.

### 2.3. Production Performance Parameters 

Body weight and feed consumption were recorded; broiler chicks were weighed at hatch, at one week of age, and at the end of every two weeks afterward until 35 days [28]. The temperature and relative humidity were recorded daily and adjusted to minimize stress surroundings in the poultry house. Mortality was recorded daily. 

### 2.4. Apparent Digestibility Coefficient 

The apparent digestibility coefficient was evaluated, using a biological assay, as affected by the different levels of ginger powder in the experimental diets. Dry matter digestibility (DMD), crude protein utilization (CPU), crude fiber utilization (CFU), and ether extract utilization (EEU) were determined according to Horwitz [29]. The apparent digestibility coefficient was estimated and expressed as a percentage [30,31].

The experimental birds were individually raised in cages and fed ad libitum on diets with ginger solution for four days as the adjusting period. During this period, the excreta was discarded. Water was available ad libitum throughout the experimental period. After the adjusting period, birds were fasted for 24 h to empty all remaining contents in the alimentary canal. In one group, birds were continuously starved for 24 h to obtain data on endogenous nitrogen excretion. Birds in the other group were force-fed with the ginger solution at a dose rate of 60 g/day/bird for three consecutive days. The excreta was collected using clean, rigid trays placed under the clean wire cages in which the birds were housed. Droppings retained on the wire screen floor of the cage were also collected. The contamination of excreta with feathers, fed, scale, and vomit was avoided. The excreta of the first group was collected after the starving period for determination of endogenous nitrogen content. Excreta of the other group was individually collected every 6 h during the three subsequent days and for approximately 24 h after the birds were fed for three subsequent days. The excreta samples were individually weighed and sprayed with 2% boric acid solution to fix the nitrogen in the samples and stored in the freezer at 20 °C until analyses. Excreta was then dried in the freeze drier for 2 days, weighed, homogenized, and ground for the estimation of nutrients. Crude protein was detected using Macrok-jeldahl method that involves the transformation of nitrogen into ammonium sulphate by acid digestion using boiling sulphuric acid. The ammonia was trapped by boric acid and was titrated using standard hydrochloric acid solution. The percentage of nitrogen was calculated from the percentage of crude protein. The crude fiber was determined using the Weende method and the Fiber Tec system. A moisture-free and fat-free sample was first digested with a weak acid solution, and then with the weak base solution. The digested residue was then collected in a filter crucible, dried and ignited. The loss of weight on ignition was the crude fiber. For crude fat determination, an organic solvent was used to extract the crude fat from a known weight of the sample. The dissolved fat was then recovered by the evaporation and condensation of the solvent. The fat extracted was a representative of the crude fat of the sample. The digestibility coefficients of the nutrients were calculated as follows:

Digestibility (%) = (NF − NE + NENC) × 100

where NF = nutrient in feed, NE = Nutrient in Excreta, and NENC = Nutrient in Excreta of negative control.

### 2.5. Antioxidant Status

Antioxidant status was investigated by measuring the antioxidant indices in the sera and livers of broiler chickens supplemented with different concentrations of ginger powder. The TSOD activity in the liver was measured using a Ransod kit from Randox Laboratories, UK, as described by Habibi, Sadeghi [32]. Liver glutathione peroxidase (Gpx) was indicated based on the protocol used by Paglia, Valentine [33]. Catalase (CAT) enzyme activity in the liver was determined using the method described by Aebi [34]. Sera MDA and total antioxidant capacity (TAC) were measured as described by Habibi, Sadeghi [32].

### 2.6. Oxidation Stability of the Feed Rations

Lipid peroxidation of the dietary feed rations, including the different concentrations of ginger powder, was determined by measuring the peroxide value (PV) and the acid value (AV) of the feed for a period of 50 days, starting from day 10. Measurements were recorded every 10 days thereafter, until day 50. The PV and the AV were measured using the methods described in AOCS [35] and Rao, Xiang [36], respectively.

### 2.7. Hematological Measurements

The samples were analyzed using a Cell-Dyn 3500 Hematology system (Abbott Laboratories, Abbott Park, IL, USA) to measure total and differential WBC and blood quality parameters, including red blood cells (RBC), hemoglobin (HGB), hematocrit (HCT), mean corpuscular volume (MCV), mean corpuscular hemoglobin (MCH), mean corpuscular hemoglobin concentration (MCHC), red cell distribution width (RDW), and platelet count (PLT).

### 2.8. Plasma Cholesterol Content 

Blood cholesterol, triglyceride, and high- and low-density lipoprotein contents were measured calorimetrically using a commercial kit from Bioassay Technology Laboratory, China. Serum was first collected by centrifugation at 2000–3000 rpm for 20 min. All reagents and standard solutions were used at room temperature. In total, 50 µL of standard solution were added to a standard well, and 40 µL of sample were added to the sample wells. Then, the corresponding LDL and HDL antibodies were added to the sample wells. Streptavidin-HRP was added to both the sample and standard wells. The plate was then incubated for 60 min at 37 °C, and 50 µL of substrate solutions A and B were added to all wells. After incubation for 10 min at 30 °C in the dark, 50 µL of stop solution were added to each well. The optical density (OD) of each well was then determined.

### 2.9. Statistical Analysis

Four experimental diets/treatments were used. For each treatment, 340 birds were randomly housed in four multi-floor batteries, each of which had five levels. Each level contained 17 birds, for 85 birds in the battery. Each level was considered as a replicate (5 replicates per treatment). Overall differences among the dietary treatments were evaluated using one-way ANOVAs via the general linear model procedure in Minitab. Differences among treatments were considered statistically different at *p* ≤ 0.05. Data were arcsine transformed before analysis to improve normality. Where significant differences occurred, pairwise Tukey post-hoc comparisons were made to identify significant differences between groups.

## 3. Results 

### 3.1. Growth Performance

All broilers appeared to be healthy, and no significant mortality occurred during the experiment. Table 2 shows the effects of different levels of ginger powder on body weight, feed consumption, feed efficiency, and weekly body weight gain of broiler chickens. The results showed that addition of ginger powder at all levels improved the body weight of the broiler chickens at 5 weeks of age (*p* < 0.001). Feed consumption of broilers fed 0, 5, 10, or 15 g/kg of ginger powder is shown in Table 2. The results showed that supplementing broiler chickens with ginger powder significantly reduced feed consumption (*p* < 0.031). Results in Table 2 showed that there was no significant effect of ginger powder on the feed efficiency of broilers. Results in Table 2 showed that the total body weight gain of birds supplemented with ginger was significantly higher than that of control birds (*p* < 0.048).

### 3.2. Apparent Digestibility Coefficient

The effects of different levels of ginger powder on nutrient digestibility are shown in Table 3. The results showed that there were significant (*p* ≤ 0.05) differences among the dietary treatment groups for DMD, CPU, CFU, and EEU. 

### 3.3. Antioxidative Indices

Table 4 shows the effect of ginger on serum and liver antioxidant indices in broiler chickens. The results in Table 4 show that broiler chickens fed diets rich in ginger powder had increased serum TAC (*p* < 0.021). However, MDA concentrations were decreased with ginger supplementation (*p* < 0.038). For the liver parameters, the results of Table 4 show that broiler chickens fed a diet supplemented with 5 g/kg ginger did not have changes in liver TSOD. However, broilers fed a diet supplemented with 10 or 15 g/kg of ginger had increased liver TSOD compared to the control group and the 5 g/kg ginger-supplemented group (*p* < 0.049). Consumption of feed rations supplemented with ginger did not affect the liver GPX level (*p* < 0.949). The concentration of CAT was increased by ginger supplementation, compared to the control group (*p* < 0.050). In contrast, broiler chickens that consumed different concentrations of ginger had decreased liver MDA (*p* < 0.020) compared to the control group.

### 3.4. Antioxidative Capacity of Feed Rations

Table 5 shows the PV of lipids extracted from stored ginger-supplemented dietary feed rations over 50 days of oxidation. From Table 5, it is evident that there was an interaction between PV value and the storage duration of ginger-supplemented feed rations. The control diet remained stable over the 50 days of storage. However, the diet supplemented with ginger at a concentration of 5 g/kg showed stability in PV value until 40 days but approached the control value by day 50. The PV value of the diet supplemented with ginger at a concentration of 10 g/kg of diet increased until day 30 but decreased to the initial value by day 40. In addition, the PV value of the feed ration supplemented with ginger at a concentration of 15 g/kg increased until day 30, started to decrease at day 40, and reached the control level at day 50 (Table 5). 

Table 6 shows the AV of lipids extracted from stored, ginger-supplemented dietary feed rations over 50 days of oxidation. The results of Table 6 also indicate an interaction between AV and storage time. The AV value increased with increasing storage duration. At all days of storage, the AV value of the control group was significantly higher than that of the groups supplemented with ginger at different concentrations.

### 3.5. Hematological Measurements

The effects of ginger powder on the blood composition of broiler chickens are shown in Table 7. The results in Table 7 show that providing broiler chickens with feed rations supplemented with ginger at 5, 10, or 15 g/kg of diet enhanced WBCs of broilers, compared to the control (*p* < 0.001). In addition, the percentages of heterophils in birds from groups fed ginger powder at 10 or 15 g/kg of diet were significantly higher than that of the control group, and lower than that of the group supplemented with ginger powder at a concentration of 15 g/kg. The results in Table 6 show that ginger supplementation had no effect on any other blood parameter.

### 3.6. Plasma Cholesterol Content 

The effects of different levels of ginger powder on blood cholesterol, triglyceride, HDL, and VLDL levels are shown in Table 8. A significant decrease in total cholesterol (TC) was observed in treatment groups supplemented with 5–15 g/kg ginger powder. A similar trend was also observed in total glycerides (TG). The HDL levels were found to be increased (*p* ≤ 0.05) in treatment groups fed 5–15 g/kg ginger powder, compared to the control group. However, LDL levels in the supplemented birds were decreased (*p* ≤ 0.05).

## 4. Discussion 

The current study was conducted to investigate the effects of increased concentrations of dietary ginger powder on the antioxidation status, production performance, and hematological parameters of broiler chickens. In this study, both the control and the treatment diets were consumed equally by chickens, indicating that the inclusion of ginger in the diet did not adversely affect the palatability of the diet. The significant increase in the total body weight gain observed in this study is in agreement with some previous studies that investigated the same variable [23,37]. However, other studies have reported that the inclusion of ginger in the diet of broiler chickens did not improve weight gain [38,39,40]. Zhang, Yang [24] investigated the effect of dried ginger root on the growth performance of broilers and found that supplementation with ginger powder led to better production performance. The positive effect of phytobiotics on the production performance of broilers was also investigated by Hashemi and Davoodi [41]. Moorthy, Ravi [42] reported that dried ginger powder increased the body weight of broilers when it was included in the diet at a level of 2%. In addition, Tekeli, Zengin [43] reported that supplementing broiler feed rations with 120, 240, and 360 ppm of ginger significantly enhanced the broiler body weight gain. Similar enhancement results in the body weight gain were reported by Onu [23], Kausar, Rizvi [44], Javandel, Navidshad [39], Herawati [37], and Ademola, Farinu [38]; however, no significant effect of ginger was observed in the average broiler daily weight gain in broilers when using 5 g of ginger/kg of diet [24] or 1 g of ginger/kg of diet [45]. In contrast, a reduction in the starter broiler growth rate was reported by Al-Homidan [46] when ginger was supplemented at a level of 60 g/kg of diet. The authors explained this reduction as a result of the toxic action of ginger. Interestingly, Zhang, Yang [24] reported a better carcass yield (*p* < 0.014) of the ginger-supplemented broilers, compared to the control group and attributed this effect to the antioxidant effect of ginger that stimulates protein and fat metabolic pathways. Conversely, Moorthy, Ravi [42] and Onu [23] suggested that supplementing broiler feed rations with ginger does not affect carcass quality.

Results of the current study showed that supplementing the broiler chickens with ginger powder reduced total feed consumption (*p* < 0.031). This result is in line with that of Herawati [37] who reported that broilers fed a 2% ginger-supplemented diet had significantly lower feed consumption than the control group. These results are in contrast with the findings of Onu [23], who reported no significant differences in the feed consumption of birds provided with different ginger treatments versus the control. In addition, Ademola, Farinu [47] observed significantly higher feed consumption in broilers fed a ginger-supplemented diet compared to the control group. 

The results of the analysis of feed efficiency showed that there were no significant differences across the treatments. This finding agrees with some studies that have investigated the same variable [25,39,48]. However, Onimisi, Dafwang [49], Moorthy, Ravi [42], Onu [23], and Onimisi, Dafwang [49] reported significantly lower feed efficiency in ginger-supplemented groups compared to the control group. These authors suggested that this outcome may be due to improved gut micro-flora, which inhibited microbial fermentation and improved feed efficiency. Conversely, Ademola, Farinu [38] reported a significant, 5% increase in feed efficiency in birds supplemented with ginger compared to control birds. 

There was a significant effect of ginger powder supplementation on nutrient digestibility in the chickens. This result could be attributed to stimulation of digestive enzymes by bioactive compounds of ginger, and thus improvement of overall digestion. The active compound gingerol contributes to the secretion of digestive enzymes, which aids in the digestive process and helps provide nutrients. The active compounds in herbs stimulate the pancreas to produce digestive enzymes in larger amounts, which leads to increased nutrient digestibility and absorption to support growth [48]. 

Biochemical studies of the blood of ginger-fed broiler chicken have previously been undertaken by several authors. Interestingly, there is a debate regarding the effect of ginger on lipid profile and blood parameters among different studies. This could be attributed to differences in strain, age, ginger level, genetics, and experiment circumstances. For example, Rehman, Durrani [50] fed broiler chickens on 10 mL of therapeutic plants (garlic, mberberine, and aloe vera)/L of drinking water alongside ginger. The authors reported that serum glucose, alanine aminotransferase, aspartate aminotransferase, and alkaline phosphatase levels were significantly reduced and the serum protein increase was significantly enhanced in supplemented broilers. Results of the same study revealed a significant reduction in the total cholesterol, triglyceride, LDL, and VLDL and significant enhancement of the HDL level in the supplemented broilers. Similarly, Zhang, Yang [24] reported that ginger inclusion increased the broiler total protein concentration and reduced cholesterol concentration at 21 and 42 days of broilers. On the other hand, Kausar, Rizvi [44] reported no effect of ginger inclusion at dosages of 2 or 4 mL/L of drinking water on serum albumin, globulin, or total protein. Al-Homidan [46] revealed a reduction in the total protein and globulin in the plasma of broilers after supplementing ginger at 60 g/kg, which could have been due to the toxic effect of ginger at that dose. However, Ademola, Farinu [47] reported that ginger supplementation at concentrations of 5, 10, or 15 g/kg did not affect total protein or albumin in the serum of broilers. 

The current effects of different levels of ginger powder on blood cholesterol, triglycerides, HDL, and LDL showed significant results. Our findings are in line with those of Shewita and Taha [17], who showed that lipid profile parameters such as total cholesterol, total triglycerides, HDL, LDL, and VLDL were found to be significantly modulated in the ginger-supplemented groups. These findings could be due to the anti-hypercholesterolemia and hypolipidemic activity of ginger. Dietary ginger acts on total serum cholesterol by inhibition of hydroxylmethyl-glutaryl-coenzyme-A reductase (HMG-CoA), or by increasing the excretion of bile acid and fecal cholesterol. However, Hayajneh [15] observed no significant differences in total protein, albumin, total cholesterol, or triglyceride levels after dietary treatment, but plasma cholesterol was found to be higher in broilers fed a diet supplemented with ginger powder. Ginger-supplemented diets administered over short terms and at low doses have been implicated in lower plasma cholesterol levels. Similarly, Barazesh, Boujar Pour [51] studied the effect of 0%, 0.5%, 1%, and 1.5% ginger powder on the blood parameters of broiler chickens. They found significant effects of ginger on blood parameters, and cholesterol and triglyceride levels. Zomrawi, Abdel Atti [52] studied the effects of 0%, 0.5%, 1%, and 1.5% ginger root powder on the blood and serum constituents of broiler chickens. They observed significant differences in serum triglyceride and cholesterol levels. In addition, inclusion of ginger root powder in the diet at levels of 0.5% and 1% lowered the cholesterol level. 

Notably, inclusion of ginger in the diet did not cause anemia in the broilers, as evidenced by the lack of a significant effect on RBC counts and hemoglobin concentration. White blood cells and their sophisticated interactions are essential for developing and stimulating immune responses [53]. Tan and Vanitha [54] concluded that essential oil constituents from the rhizomes of *Z. officinale* exert immune-stimulating effects by enhancing the phagocytic activity of heterophils. Furthermore, Vattem, Lester [55] found that dietary supplementation of *Zingiberaceae* spices significantly increased the number and viability of coelomcytes, in addition to promoting differentiation into neutrophil-like cells, thus increasing phagocytic activity. Ginger consumed at a level of 100 mg/kg of diet was found to be effective for stimulating innate immunity by increasing the phagocytic capacity of heterophils, and for humoral immunity by increasing the production of antibodies; consequently improving the immunological profile of broiler chickens [56]. Additionally, Azhir, Zakeri [57] found that adding ginger rhizome powder at a concentration of 10 g/kg improved the humoral immunity of broilers at 35 days of age. Nidaullah, Durrani [58] observed that an aqueous extract of ginger rhizome mixed with water acted as an immune stimulant against Newcastle disease and coccidiosis. Ademola, Farinu [38] observed that ginger provided to chickens at a concentration of 1.0% caused a significant decrease in the total number of WBCs. The authors also found that ginger failed to affect the RBCs of broiler chickens. According to Nasiroleslami and Torki [59], the differential count of WBCs was similarly not affected by the dietary inclusion of ginger essential oil. In the current study, the inclusion of ginger in the diet of broiler chickens did not affect the hematological parameters of the birds, except for the total WBC and percentage of heterophils. The total WBC count increased significantly with an increased level of ginger in the diet. This indicates an enhanced immune response of cells involved in the innate and/or specific immune system.

Oxidative stress in broilers is associated with a high concentration of MDA and fatty acid peroxidation, due to increased free radicals [60]. Ginger has been shown to enhance antioxidative status. The results of the current study are in agreement with those reported in the literature. For example, Safiullah, Chand [61] reported that the inclusion of ginger powder and ginger essential oil in the feed rations of broiler chickens decreased MDA in liver and sera samples compared to birds fed a control diet. Wen, Gu [20] reported that the addition of ginger extract significantly increased the total antioxidant potential, decreased MDA content, and increased glutathione peroxidase activity in the serum and breast muscles. Interestingly, Mountzouris, Paraskeuas [62] showed that inclusion of a phytogenic premix including ginger and other natural herbs elevated the expression of cytoprotective genes against oxidation. In particular, the cytoprotective genes opposing oxidation were upregulated generally in the duodenum and ceca, and secondarily in the jejunum [62].

## 5. Conclusions 

Ginger inclusion in the broiler diet can be safely used to enhance the production performance, immune response, and antioxidative status of broiler chickens.

## Figures and Tables

**Table 1:** Formulation and chemical analyses of the basal broiler diets.

Ingredient%		Starter	Grower	Finisher
		0–7 Day	8–21 Day	22–35 Day
Corn		55.6	57.6	61.2
Soyabean Meal		39.4	35.6	32.25
Soya oil		1.35	3.2	3.35
Limestone		1.45	1.45	1.3
DiCalcium Phosphate		1.4	1.4	1.2
Salt		0.21	0.21	0.2
L-Lysine		0.12	0.12	0.1
DL-Methionine		0.27	0.27	0.26
Vitamin-Mineral Premix *		0.2	0.2	0.2
Total%		100	100	100
Nutrient Composition				
Chemical Analysis				
Crude Protein (CP) (%)		22	20	18
Metabolizable Energy (kcal/kg)		2975	3025	3150
Fat (g/kg)		3.86	5.75	6
Calculated analysis				
Calcium (g\kg)		0.9	0.84	0.76
Phosphorus (g\kg)		0.45	0.42	0.38
Sodium (g\kg)		0.19	0.19	0.19
Lysine (g\kg CP)		1.22	1.12	0.97
Methionine (g\kg CP)		0.46	0.45	0.4
Choline (mg\kg)		1420	1329	1260
Proximate analysis of the ginger used				
Crude protein	6.0			
Ash	3.0			
Crude fiber	3.0			
Dry matter	91.2			

* Supplied per kg of premix: trans-retinol (A), 12,500,000 IU; cholecalciferol (D3), 500,000 IU; α-tocopherol acetate (E), 75,000 mg; thiamine (B1), 4500 mg; riboflavin (B2), 8000 mg; pyridoxine (B6), 5000 mg; vitamin B12, 22,000 mg; pantothenic acid, 20,000 mg; folic acid, 2000 mg; biotin, 200,000 μg; Fe, 100,000 mg; Co, 250 mg; Mn, 100 mg; Cu, 10,000 mg; Zn, 80,000 mg; I, 1000 mg; Se, 300 mg; Mo, 0.5 mg; Ca, 7.7%; P, 0.01%; Na, 0.18%.

**Table 2:** The effects of different levels of ginger powder on body weight, feed consumption, feed efficiency, and weekly body weight gain of broiler chickens.

	Treatment (Ginger g/kg)					
Age	0	5	10	15	SEM	*p*-Value
Bodyweight (g)						
1 day	43	42	42	43	0.000	−
1 week	99	108	107	97	11.21	<0.213
2 week	280	287.5	287.9	283	8.98	<0.342
3 week	640 ^a^	715.5 ^b^	679.9 ^b^	665 ^b^	4.13	<0.001
4 week	1090 ^a^	1205.5 ^b^	1110.9 ^b^	1120 ^b^	15.53	<0.001
5 week	1680 ^a^	1792.5 ^b^	1690.9 ^b^	1730 ^b^	9.85	<0.001
Feed Consumption (g)						
1 week	72.7	67.4	66.2	67.8	1.64	<0.060
2 week	210	215	230	211	2.56	<0.341
3 week	620.9 ^a^	614.2 ^b^	590.5 ^b^	580.8 ^b^	12.21	<0.001
4 week	950	953	920	934	15.31	<0.215
5 week	1410.5 ^a^	1281.5 ^b^	1280.6 ^b^	1354.1 ^b^	36.72	<0.050
Feed Efficiency						
1 week	1.9	2.3	2.1	2.4	0.14	<0.118
2 week	1.09	1.12	1.12	1.15	0.98	<0.213
3 week	1.4	1.5	1.3	1.4	0.05	<0.409
4 week	1.11	1.13	1.13	1.2	0.18	<0.134
5 week	1.2	1.2	1.1	1.2	0.13	<0.079
Weekly Body Weight Gain (g)						
1 week	56 ^a^	66 ^b^	65 ^b^	54.0 ^a^	2.65	<0.025
2 week	181	179.5	180.9	186	6.84	<0.642
3 week	360 ^a^	428 ^b^	392 ^b^	382.0 ^b^	11.75	<0.001
4 week	450	490	431	455	23.12	<0.216
5 week	590	587	580	610	18.88	<0.06
Total Body Weight Gain (g)	1637	1750.5	1649	1687	9.45	<0.048
Total Feed Consumption (g)	3264	3131	3087	3148	12.10	<0.031

Means within rows with different letters are statistically different at *p* ≤ 0.05, SEM = standard error of the mean, calculated by one-way analysis of variance (ANOVA) and the general linear model procedure of Minitab.

**Table 3:** The effects of different levels of ginger powder on nutrient utilization coefficients in broilers.

Treatment (Ginger g/kg)						
	Control	5	10	15	SEM	*p*-Value
DMD	58.6 ^a^	73.9 ^b^	71.1 ^b^	77.9 ^c^	2.00	<0.001
CPU	60.5 ^a^	74.3 ^b^	76.1 ^b^	73.9 ^b^	5.00	<0.001
CFU	64.8 ^a^	73.9 ^b^	74.2 ^b^	73.5 ^c^	3.80	<0.001
EEU	58.2 ^a^	65.6 ^b^	65.8 ^b^	64.9 ^b^	4.62	<0.050

All treatment groups received a soybean basal diet, means within rows with different letters are statistically different at *p* ≤ 0.05, *n* = 5 Dry matter digestibility (DMD), crude protein utilization (CPU), crude fiber utilization (CFU), and ether extract utilization (EEU).

**Table 4:** Antioxidant parameters in serum and livers of broiler chickens fed various concentrations of ginger powder.

Treatment (Ginger g/kg)						
Parameter	0	5	10	15	SEM	*p*-Value
Serum						
TAC (mmol/L)	0.6 ^a^	0.9 ^b^	1.1 ^b^	0.9 ^b^	0.03	<0.021
MDA (nmol/mL)	4.1 ^a^	2.9	2.8 ^b^	3.0 ^b^	0.05	<0.038
Liver						
TSOD (U/mg pro.)	4.0 ^a^	3.9 ^a^	4.5 ^b^	4.9 ^b^	0.95	<0.049
Gpx (U/mg pro.)	0.6	0.5	0.7	0.6	0.01	<0.949
CAT (k/mg pro.)	0.2 ^a^	0.6 ^b^	0.7 ^b^	0.7 ^b^	0.11	<0.050
MDA (nmol/mg pro.)	6.1 ^a^	5.0 ^b^	4.2 ^b^	4.0 ^b^	0.20	<0.020

Means within rows with different letters are statistically different at *p* ≤ 0.05, *n* = 5. SEM = standard error of the mean, calculated by one-way analysis of variance (ANOVA) and the general linear model procedure of Minitab. TAC = antioxidant capacity; TSOD = total superoxide dismutase; Gpx = glutathione peroxidase; CAT = catalase; MDA = malondialdehyde.

**Table 5:** Peroxide value (PV) of lipid extracted from stored ginger-supplemented dietary feed rations over 50 days of oxidation.

Peroxide Value (mEq/kg)						
Treatment (Ginger g/kg)						
Time (Days)	0	5	10	15	SEM	*p*-Value
10	16.11 ^a^	20.02 ^b^	22.09 ^b^	17.01 ^a^	2.02	<0.001
20	20.00 ^a^	28.01 ^b^	30.41 ^b^	22.51 ^a^	2.99	<0.001
30	19.52.^a^	30.89 ^b^	40.74 ^c^	40.19 ^d^	2.84	<0.001
40	18.63.^a^	25.22 ^b^	30.32 ^b^	30.55 ^c^	2.01	<0.001
50	19.51 ^a^	21.03 ^a^	25.42 ^b^	22.84 ^a^	3.15	<0.001

Means within rows with different letters are statistically different at *p* ≤ 0.05, *n* = 5. SEM = standard error of the mean, calculated by one-way analysis of variance (ANOVA) and the general linear model procedure of Minitab.

**Table 6:** Acid value (AV) of lipid extracted from stored ginger-supplemented dietary feed rations over the time of oxidation.

Acid Value (mg KOH/g)						
Treatment (Ginger g/kg)						
Time (Days)	0	5	10	15	SEM	*p*-Value
10	10.22 ^a^	6.02 ^b^	7.01 ^b^	7.99 ^b^	4.01	<0.001
20	18.04 ^a^	10.65 ^b^	11.21 ^b^	10.45 ^b^	3.59	<0.001
30	24.05 ^a^	18.54 ^b^	20.01 ^b^	15.22 ^c^	2.32	<0.001
40	30.14 ^a^	22.01 ^b^	19.87 ^b^	19.54 ^b^	3.91	<0.001
50	12.44 ^a^	14.01 ^b^	13.98 ^b^	12.45 ^b^	4.00	<0.001

Means within rows with different letters are statistically different at *p* ≤ 0.05, *n* = 5. SEM = standard error of the mean, calculated by one-way analysis of variance (ANOVA) and the general linear model procedure of Minitab.

**Table 7:** Hematological and biochemical parameters of broilers fed ginger powder.

	Treatment (Ginger g/kg)					
Parameter	0	5	10	15	SEM	*p*-Value
WBC (K/uL)	47.9 ^a^	79.6 ^b^	75.3 ^b^	74.2 ^b^	4.42	<0.001
Heterophils (%)	13.1 ^a^	24.6 ^b^	29.3 ^b^	32.6 ^c^	1.26	<0.001
Lymphocytes (%)	72.3	73.9	68.2	65.4	4.06	<0.474
Monocytes (%)	9.2	7.6	11.03	5.84	1.82	<0.256
Eosinophils (%)	0.03	0.02	0.04	0.01	0.01	<0.318
Basophils (%)	2.9	2.4	4.15	2.5	0.76	<0.384
RBC (M/uL)	2.9	2.2	2.41	2.00	0.27	<0.469
HGB (g/dL)	13.2	13.3	12.26	12.7	0.51	<0.266
HCT%	34.7	34.7	31.28	34.6	1.09	<0.104
MCV (fL)	127.8	130.4	129.8	128.0	1.87	<0.702
MCH (pg)	49.9	51.7	50.9	50.2	0.64	<0.238
MCHC (g/dL)	39.0	39.6	39.3	39.2	0.64	<0.918
RDW%	12.1	12.3	12.1	12.4	0.45	<0.975
Thrombocyte PLT (K/uL)	1.79	3.7	2.1	0.8	1.05	<0.306

Means within rows with different letters are statistically different at *p* ≤ 0.05, *n* = 5. SEM = standard error of the mean, calculated by one-way analysis of variance (ANOVA) and the general linear model procedure of Minitab. WBC = white blood cells; RBC = red blood cells; HGB = hemoglobin; HCT = hematocrit; MCV = mean corpuscular volume; MCH = mean corpuscular hemoglobin; MCHC = mean corpuscular hemoglobin concentration; RDW = red cell distribution width; PLT = platelet count.

**Table 8:** The effects of different levels of ginger powder on blood cholesterol, triglyceride, high- and low-density lipoprotein content in broiler chickens.

Treatment (Ginger g/kg)						
	Control	5	10	15	SEM	*p*-Value
TC, mg/dL	160 ^a^	145 ^b^	140 ^b^	121 ^c^	8.00	<0.001
TG, mg/dL	40.0 ^a^	28.5 ^b^	25.2 ^b^	22.8 ^b^	5.5	<0.001
HDL, mg/dL	69.8 ^a^	83.0 ^b^	90.9 ^b^	92.9 ^b^	7.9	<0.001
LDL, mg/dL	60.7 ^a^	29.9 ^b^	27.8 ^b^	30.1 ^b^	5.6	<0.001

All treatment groups received a soybean basal diet. Means within rows with different letters are statistically different at *p* ≤ 0.05, *n* = 5, total cholesterol (TC), triglyceride (TG), high-density lipoprotein (HDL), and low-density lipoprotein (LDL).

## Data Availability

No new data were created or analyzed in this study. Data sharing is not applicable to this article.
